# Consumer Decision-Making Creativity and Its Relation to Exploitation–Exploration Activities: Eye-Tracking Approach

**DOI:** 10.3389/fpsyg.2020.557292

**Published:** 2021-01-12

**Authors:** Eunyoung Choi, Cheong Kim, Kun Chang Lee

**Affiliations:** ^1^SKK Business School, Sungkyunkwan University, Seoul, South Korea; ^2^Economics Department, Airports Council International World, Montreal, QC, Canada; ^3^Department of Health Sciences and Technology, Samsung Advanced Institute for Health Sciences and Technology, Sungkyunkwan University, Seoul, South Korea

**Keywords:** creative consumer decision-making, eye-tracking, exploratory activity, exploitative activity, time constraints, task difficulty

## Abstract

Modern consumers face a dramatic rise in web-based technological advancements and have trouble making rational and proper decisions when they shop online. When they try to make decisions about products and services, they also feel pressured against time when sorting among all of the unnecessary items in the flood of information available on the web. In this sense, they need to use consumer decision-making creativity (CDMC) to make rational decisions. However, unexplored research questions on this subject remain. First, in what ways do task difficulty and time constraints affect visual attention on exploitative and exploratory activities differently? Second, how does the location of the reference (i.e., hints) influence the level of visual attention to exploitative and exploratory activities depending on affordance theory? Third, how do exploratory and exploitative activities affect CDMC? Eye-tracking experiments were conducted with 70 participants to obtain relevant metrics such as total fixation duration (TFD), fixation count (FC), and visit count (VC) to answer these research questions. Our findings suggest that task difficulty influences exploitative activity, whereas time constraint is related to the exploratory activity. The result of the location of hints aligns with the affordance theory for the exploitative activity. Besides, exploratory activity positively affected CDMC, but exploitative activity did not show any effect.

## Introduction

Over the past decade, the concept of web-based product information has been overwhelmingly dominating the manufacturing industry as an essential tool for customer engagement. The overarching philosophy of web information is providing an interactive and enlightening resource to induce consumers to make a favorable purchase decision on products. However, this concept also brought problematic decision-making issues to both consumers and firms.

From the perspective of customers, because of the flood of information attributable to the digital revolution, it is imperative for customers to recognize correctly the benefits of the products they need among a variety of selections and then to make rational and creative decisions (Leeflang et al., 2014). However, the increase in consumer awareness and technological sophistication has made it increasingly difficult for them to make rational purchasing decisions (Kulshreshtha et al., 2017). Typical consumers have busy lives and are time-constrained. Thus, they have difficulties in spending the considerable time required to make rational and appropriate choices. In this sense, they need to make creative decisions about the set of possible product attributes under extreme time pressure and conditions of selective overload (Reutskaja et al., 2011).

Meanwhile, as a vast range of similar products is introduced every day, competition in today’s durable web markets has thus become quite intense. That being said, providing an informative, creative decision-making basis to consumers became a much more important activity that manufacturers utterly need to consider. However, because of the dynamic nature of consumer behavior (Seiler, 2013; Habibi et al., 2016), it would not be easy to come up with a strategic direction to induce the customers’ creativity. In other words, discovering consumers’ insights that could help enhance their creativity has recently forced manufacturers and brand managers to pursue productive and sustainable marketing strategies, launch new products, improve product quality, and implement new technologies (Kim and Han, 2014; Chung and Lai, 2017; Kim et al., 2020b).

Understanding how customers become creative in their decision-making using provided information would be necessary for conducting the design of website structures. Hence, field practitioners of manufacturing companies have worked together for a long while to identify and satisfy customers’ creative decision-making to remain competitive in target markets. In this sense, it could be said that firms’ business problem-solving to support consumers’ creativity has been accumulating for a considerable amount of time and effort. When such creativity is used to its maximum to define and resolve customers’ problems from their perspectives, firms’ strategic goals in marketing may be accomplished with great success.

Previous studies have supported the positive effect of creativity on business success (Amabile, 1996; Perry-Smith, 2006; Althuizen and Reichel, 2016; Baack et al., 2016) from the perspective of corporations. Also, the individual-level creativity has been a motivating research topic in the field of psychology and business for a while (Shalley, 1991; Bharadwaj and Menon, 2000; Taggar, 2002; Pirola-Merlo and Mann, 2004; Hirst et al., 2009). Considering the importance as well as a substantial amount of time and effort investment of firms in consumers’ creativity for decision-making, figuring out how to adequately induce consumers to utilize their creativity is crucial. However, the former research mostly emphasized the organization-level creativity that could facilitate insiders’ creativity to enhance firms’ business competitiveness (Amabile, 1996; Perry-Smith, 2006; Althuizen and Reichel, 2016; Baack et al., 2016), whereas few have investigated the importance of consumer creativity in decision-making (Rosa et al., 2014). Also, most of these studies employed self-report surveys that could be problematic (Farh and Dobbins, 1989). There could also be a strong likelihood of common methods bias when respondents report creativity using the self-report approach (Spector, 2006). Therefore, in this research, we conducted rigorous consumer-oriented creativity research that could advance theoretical perspectives, as well as provide practical implications that firms could apply to customer decision-making with creativity. By doing so, this study will be able to fill the gap by suggesting fruitful findings in the field of consumer creativity research that the previous research did not discover without facing a risk of bias, which might occur from using the self-report instrument.

**FIGURE 1:**
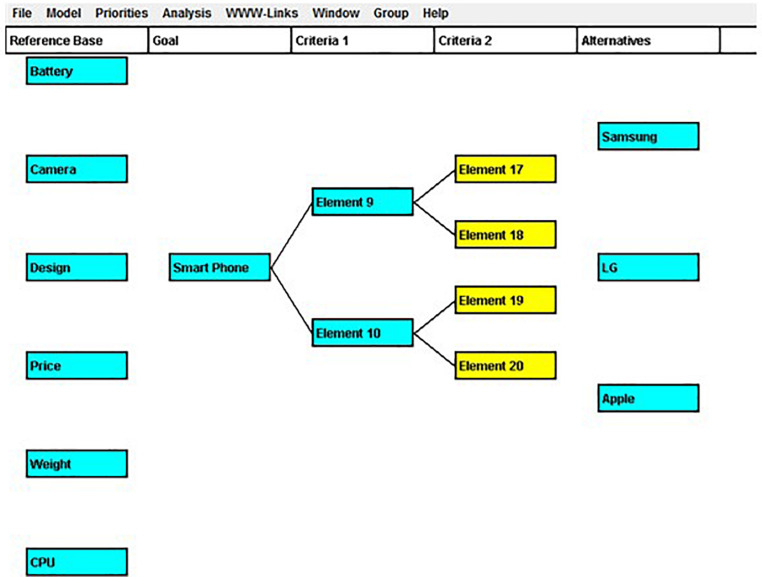
Problem presentation window for decision-making.

This study adopted an eye-tracking approach in order to measure customer creativity in the processing of web information of smartphone products that measured such indices as total fixation duration (TFD), fixation count (FC), and visit count (VC), to investigate the way in which consumers’ visual attention is associated with consumer decision-making creativity (CDMC) and the way in which exploitative and exploratory activities differ, depending on time constraints and task difficulty. By applying an eye-tracking approach, we could avoid possible bias that occurred from a self-report survey and derived more feasible outcomes. As a stimulus of the visual attention, we employed a java-based decision-making software, Web-HIPRE (Mustajoki and Hämäläinen, 2000), which derives to value-focused thinking (Turunen et al., 2018) (Figure 1). The outcomes of the participants on the web-HIPRE were also used in the CDMC assessments.

We addressed three specific research questions in this study: first, how do task difficulty and time constraints affect visual attention differently? Second, how does the location of the reference (i.e., hints) influence the level of visual attention to exploitative and exploratory activities, respectively? Third, does the notion of visual attention affect CDMC?

## Literature Review and Hypotheses

### Exploitation vs. Exploration: Task Difficulty and a Time Constraint

The exploitation–exploration framework divides learning patterns broadly according to two aspects. In the definition of March (1991), exploration involves terms such as search, variation, risk-taking, experimentation, play, flexibility, discovery, and innovation, whereas exploitation includes such things as refinement, selection, production, efficiency, implementation, and execution. In particular, exploration would be riskier, with a higher possibility of failure, but it would bring more responsive and adaptive to turbulent market environments. Besides, exploitation is considered to be less risky and provide quicker returns, but it would be self-destructive in the long run. Citing Levinthal and March (1993), engaging in exploration entails the pursuit of new knowledge, of things that might come to be known, whereas engaging in exploitation involves the use and development of things known already.

At the individual level, exploitation is related to high-level engagement designed to optimize the performance of a current task; in contrast, exploration involves disengaging from the current task to experiment with new ideas that may result subsequently in radical innovations (Laureiro-Martínez et al., 2015). Exploitation may be more useful when reacting to current environmental needs, compared with exploration because exploitation is associated with speedy and precise production. On the other hand, the outcomes of exploration have a longer time horizon and may even be less certain. Henceforth, exploration is recommended for long-term projects (March, 1991; Laureiro-Martínez et al., 2015).

From the perspective of CDMC, consumers’ exploitation utilizes existing knowledge to make a quick and relatively more straightforward decision; for instance, when they are buying a well-known product or already possess enough information about the product. Conversely, exploration will be actively employed when consumers find it difficult to finalize making a purchase decision because intrinsic knowledge is insufficient, thus requiring additional knowledge from the exogenous source. However, when it comes to reality, both activities would be compulsory in the process of CDCM for specifically smartphone purchasing as a case in this research because most of the brand new smartphones are generally launched with novel functionality in terms of both hardware and software as well as features that consumers are already familiar with. Therefore, the combination of exploitation and exploration will continuously and naturally occur during the process of CDMC in innovative product purchasing, including smartphones.

Studies that address exploitation–exploration with eye-tracking methods are rare, although Beesley et al. (2015) conducted one such study. The authors’ designated participants to play the role of a scientist who discovered a new chemical that could create a mutant organism, and the task was to design an experiment that predicted what mutations would occur when certain chemical pairs were mixed. They stated that uncertainty-based exploratory processes were more sensitive to contextual changes between training stages than were predictive-based exploitative processes. Another result of their experiment is that both exploitation and exploration had an influence on the attention participants paid to stimuli during associative learning; the exploitative attention process affected novel learning, whereas the exploratory attention process yielded results that are beneficial to learning.

There have been considerable studies on exploitation–exploration and creativity (Audia and Goncalo, 2007; Miron-Spektor et al., 2011; Seo et al., 2015) and also on creativity, task difficulty (Friedman and Förster, 2001; Chae et al., 2015), and time constraints. We based our research on the following antecedent studies: (1) assessment of a website’s complexity and difficulty using eye tracking (Wang et al., 2014) and (2) consumer decision-making under time pressure measured with similar metrics (Reutskaja et al., 2011). Greater task difficulty and time constraints would put consumers into more predicament. Therefore, we presume that participants would do more exploitative activities that might be safer and promise quicker returns with greater difficulty and given time constraint, whereas they would do more exploratory activities with lower difficulty and without time constraint. Thus, we performed eye-tracking experiments to test the following hypotheses related to exploitation–exploration and task difficulty, as well as exploitation–exploration and time constraints.

H1. Visual attention on exploitative activity will increase with a more difficult task, whereas visual attention on exploratory activity will increase with an easier task.

H2. Visual attention on exploitative activity will increase with a time constraint, whereas visual attention on exploratory activity will increase without time constraint.

### Exploitation vs. Exploration: The Location of Information

The concept of affordability, which refers to attributes that can be executed between the world and actors (people or animals), was introduced in the book of Gibson (1979), *The Ecological Approach to Visual Perception*. It is defined as the nature of animal–environment interactions that determine behavioral outcomes. In other words, the question is explained by Affordance Theory based on the claim that familiar information or situations influence a person’s behavior (Gibson, 1979; Michaels, 1988; Bub et al., 2018). The concept of affordance applied in our study took a different conceptual approach to that of Gibson and borrowed from Norman’s Perceived Affordance Theory. In 1988, Norman began using the concept of affordance in his book, *The Psychology of Everyday Things* (POET), with respect to human–computer interaction (Norman, 1988). Norman introduced this term in the field of design to define specific attributes of physical artifacts that help us understand the way in which ergonomics should be manipulated. However, he later defined his theory as perceived affordance to differentiate it from real affordance because of the improper use of the term (Norman, 1999). Eichelman (1970) also discovered the effect of familiarity for simultaneous matching tasks. This notion signifies that familiar information and circumstances affect human behavior through experience or certain innate abilities, and people assess and respond to stimuli according to their perceptible attributes (Zhao et al., 2013; Boy et al., 2016).

These findings from the previous studies suggest that the concept of familiarity could be employed as a critical mass to decide the location of crucial information of products on web sites, such as a hint for discount, which might induce consumers to derive decision-making creativity. In that sense, exploitative activities would be significantly related to familiarity because exploitation is considered as improving and refining existing competencies and ideas. Employing what Atuahene-Gima (2005) stated, the exploitation of formulations with common ingredients extends current knowledge and seeks greater efficiency and improvements. When it comes to the location of web-based information, consumers conducting exploitative activities would be able to find it much adequate with familiar spots, for instance, the left side of the screen where all the filters and menus are generally located (Shrestha and Lenz, 2007).

Exploration, on the other hand, accompanies experimentation with new subjects and areas. Also, exploration entails the development of new knowledge through experimentation that fosters the variation and novelty needed for more radical changes (Laureiro-Martínez et al., 2015). In other words, exploration would require knowledge, habit, and behavior that are unfamiliar and more provocative compared to exploitation. In contrast to exploitation, consumers would go far beyond their familiarity when they do exploratory activities on the web-based information; thus, location familiarity of web information would not have any significant impact on their creative decision-making procedures.

According to Natraj et al. (2015), eye tracking has the advantage of being able to assess directly the effect of temporally assigning visual and spatial interests. Therefore, eye tracking would be a useful tool to reveal where vital information should be located on web pages. Based on these theoretical backgrounds, the following hypotheses were proposed to find out the relationship between the location of information, exploitation, and exploration.

H3a. When the hint position is located on the left side rather than the right side, visual attention in exploitation activity will be more concentrated.

H3b. Hint location will not affect the concentration of visual attention in exploration activity.

### Creativity and Visual Attention

According to previous research on the subject since Guilford’s groundbreaking presidential address to the American Psychological Association in 1950 (Guilford, 1950), numerous definitions of creativity in business have been proposed. Then, these definitions gradually have become more sophisticated (Perry-Smith, 2006; Yeh et al., 2016). Many past researchers have paid significant attention to the positive effects of creativity (Goldenberg et al., 1999; Smith and Yang, 2004; Baack et al., 2016) and analyzed ways in which to enhance it (Suh et al., 2010; Bai et al., 2016; Yang and Yang, 2016). It is because the positive effect of creativity is an underlying source of innovation that can catalyze an organization’s growth (Woodman et al., 1993). Also, studies of creativity have advanced in various areas, including business, social science, and engineering, among others. Consumers’ creativity in this study employed the arguments of Guilford (1965) and Hirschman (1980) that productive thinking to generate the solution during the process of decision-making in purchasing a product on the website using relevant and adequate information provided. Hirschman (1980) also suggested that creativity is essential during the problem-solving process; thus, consumers’ creativeness would be the fundamental component for making a decision in the purchasing progression. However, relatively less attention has focused on studying CDMC.

The eye-tracking technique has been adopted to examine human visual attention based on the eye–mind assumption (Just and Carpenter, 1980). In general, the location on which the eye fixates reflects attention, whereas fixation duration reflects processing difficulty and amount of attention (the longer the information is fixated, the more complex it is or the more deeply it is processed). Specifically, fixation duration varies depending on the type of information (e.g., text vs. graphics) and type of task (e.g., reading vs. problem-solving). Further, fixation locations and duration reflect the individuals’ reading strategies and prior knowledge or experience (Hyona et al., 2002). Besides, scan path patterns indicate the cognitive strategies individuals use in goal-oriented tasks (Gandini et al., 2008). Thus, eye tracking has been found to be a useful method in psychology to study cognitive processes, largely because of the assumed link between attention and what we look at Rothkopf et al. (2007). Eye movements are associated closely with shifts in attention—in that our eyes may be drawn unconsciously to something of interest, and our attention follows, or we may choose to look at something to direct our attention to it. A variety of research supports this idea that changes in fixation reflect changes in the focus of our attention (Lee and Ahn, 2012).

Eye movement often is used in creativity research because the eye tracker can track eye dynamics precisely and navigate the internal cognitive processes that cannot be seen in overt behavior (Huang, 2017). According to Yeh et al. (2014), the amount of attention, eye movement, and working memory interact to affect problem resolution. Drawing on these previous studies, we proposed the following hypotheses on the relations between exploitation–exploration, creativity, and the notion of visual attention.

H4. The greater the visual attention on exploratory work, the higher the CDMC will be.

### Web-HIPRE

Web-HIPRE (HIerarchical PREference analysis on the World Wide Web) (Mustajoki and Hämäläinen, 2000) is a java-based software for multicriteria decision analysis tool. It is based on HIPRE 3+ (Hämäläinen and Lauri, 1995), a well-known software of decision-making support system (Turunen et al., 2018). It has been frequently used as an essential tool for multicriteria decision analysis that is harnessed for creative problem-solving (Geldermann et al., 2009; Vacik et al., 2014). It also provides an implementation of multiattribute value theory (Keeney and Raiffa, 1993) and the analytic hierarchy process (AHP) (Saaty, 1990, 1994; Salo and Hämäläinen, 1997) to support the different phases of decision analysis such as structuring of the problem (French et al., 1998), prioritization, and analyzing the results (Mustajoki and Hämäläinen, 2000).

## Materials and Methods

This study was conducted with the approval of Sungkyunkwan University, in compliance with the guidelines and regulations of the university institutional review board (IRB no. 2017-12-011-022) for the method.

### Participants

A total of 80 physically and mentally healthy undergraduate students from Sungkyunkwan University in Seoul were recruited to participate in two experiments, which have different conditions in task difficulty and time constraint. The participants were selected randomly to avoid the error that may occur because of an unequal distribution of grades and majors. Among them, a total of 70 data points (33 for task difficulty and 37 for time constraint studies) were analyzed, excluding 10 participants: four extreme outliers and six unsuccessful eye-tracking calibrations. Thirty-eight of the 70 participants were females: 20 in the task difficulty group and 18 in the time constraints group. Participants’ mean age was 23.16 [standard deviation (SD) = 1.78]. The Web-HIPRE screen with area of interest (AOI)–Reference on the left side was assigned to 15 participants for the task-difficulty experiment and 17 people for the time-constraint experiment. The easy task was assigned to 17 of 33, and the time-constraint option was applied to 18 of 37 (Table 1). We ensured that no one had participated previously in a similar eye-tracking experiment and instructed participants to avoid excessive drinking or lack of sleep before taking part in the experiment. In order to obtain more precise eye-tracking data, the participants were asked not to wear contact lenses and excluded those who had severe astigmatism.

**TABLE 1:** The distribution of the participants for task difficulty, time constraint, and hint location.

	Task difficulty		Time constraint		Sum
Manipulation	Easy	Difficult	Yes	No	
	17	16	18	19	70

Hint location	**Left**	**Right**	**Left**	**Right**	

	15	18	17	20	70

### Procedure

Upon arrival, participants were asked to fill out a questionnaire that included demographic information. In addition to the questionnaire, a detailed explanation about the experimental procedure and the tracking device was provided, including a device-mounted eye tracker that would record their eye movements unobtrusively. After participants agreed, the calibration process was carried out. Before performing the task, they watched a training video that guides how to use Web-HIPRE. The formal experiment began after confirming that the participants had a good understanding of the procedure.

**FIGURE 2:**
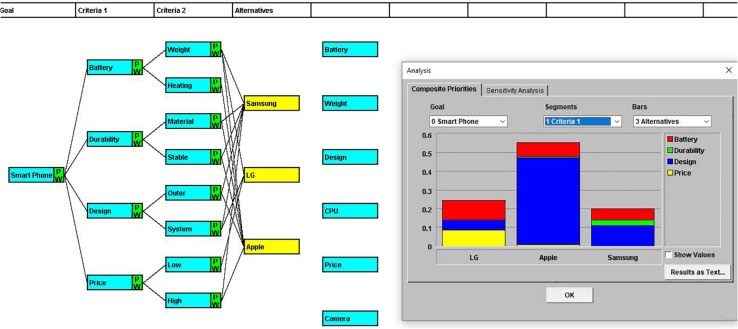
An outcome of decision-making through Web-HIPRE task.

**FIGURE 3:**
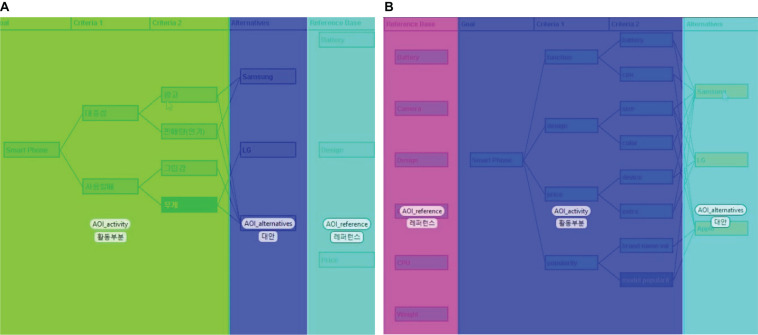
Windows that show easy **(A)** and hard **(B)** tasks with three AOIs. **(A)** Easy task locating reference base on the right side. Note 1: AOI-Activity is visible at the left, and AOI-Alternatives is illustrated in the middle. Three hint nodes are given in the AOI-Reference on the right: battery, design, and price. Note 2: The participant created two nodes in Criteria 1, popularity, and ease of use. The subattributes were organized as follows: popularity advertisement and sales volume, and ease of use grip, and weight. **(B)** Hard task locating the AOI-Reference on the left side. Note 1: AOI-Activity is visible from the middle, and AOI-Alternatives is illustrated at the right. Six hint nodes are given in the AOI-Reference on the left: battery, camera, design, price, CPU, and weight. Note 2: The participant created four nodes in Criteria 1, function, design, price, and popularity. The subattributes were organized as follows: function-battery and CPU, design-size and color, price-device and extra, and popularity-brand name value and model popularity.

Before starting the eye-tracking experiment, the participants’ eye points were calibrated using Tobii studio software (version 3.3, Tobii Technology) to ensure that measurements would be made accurately and precisely on the experiment’s AOIs. They were requested to sit at a distance of 65 cm from the screen with minimal movement as possible. Thereafter, the visual attention data were recorded while the participants developed a decision-making model by harnessing the Web-HIPRE program. Thereafter, the eye tracker measured their eye movements while the participants developed a decision-making model using the Web-HIPRE program. Participants were given the following decision-making tasks: “Let’s assume you are buying a smartphone. Which product would you buy from Samsung, LG, and Apple?” Then the participants were presented with the screen shown in Figure 1. To address this decision-making problem, participants worked on Web-HIPRE to configure criteria to be applied, supposing they purchase a smartphone model based on their preferred attributes. The reference base menu for participants included six categories: batteries, cameras, designs, prices, weight, and CPU, which are used widely in smartphone comparisons. Participants would implement a primary decision-making strategy by deciding whether to exploit the reference base menu in the decision-making process. The Web-HIPRE automatically presents the final decision result based on the criteria suggested by the experiment participants (Figure 2). The Web-HIPRE screen consists of three AOIs: reference, task activity, and alternatives as marked with AOI-reference, AOI-activity, and AOI-alternatives in Figure 3, respectively.

### Manipulations

#### Stimulus: Web-HIPRE

For this experiment, we harnessed the Web-HIPRE^1^, which is a decision support system (DSS) developed by the Systems Analysis Laboratory of Aalto University’s School of Science and Technology. It is used as an AHP for decision support (Mustajoki and Hämäläinen, 2000) and is the first web-based DSS software with multicriteria (Mustajoki et al., 2004). The software helps users make informed decisions by developing decision-making models and processing the prioritization of each option. The experimental paradigm included watching a video on how to use the DSS software, Web-HIPRE with the following instruction:

“You can set 4 top comparison criteria (2 for easy task participants) in Criteria 1, and create two subelements for each element belonging to Criteria 1. (Therefore, the number of elements in Criteria 2 is all eight. For easy task participants, the elements in Criteria 2 are all four.) At this time, double-click to create element boxes. To change the name, click once, and press Enter key. After creating elements, you need to connect the parent and child items. To connect, click the parent item to the left and right-click the child item. Once all the criteria have been linked, you can evaluate each alternative according to the eight (or four) comparative items in Criteria 2. When writing, you can use the items in the reference base or produce the new standard. After completing the connection, you need to set the priority. To determine the priority, go to the ‘AHP’ submenu of the ‘Priorities’ menu. Pairwise comparisons options are given, and you can weigh them according to how vital their attributes are to your decision. When all settings are completed, you can check the result by visiting the ‘Composite Priorities’ submenu in the ‘Analysis’ menu” (Figure 2).

#### Task Difficulty

In this study, we compared the level of CDMC and the results of eye tracking between two groups, one of which performed a simple task, whereas the other engaged in a more difficult one. Task difficulty was assessed by applying the innovative idea of Wang et al. (2014), which the manipulations were related directly to the complexity of the selection options in the task areas of the Web-HIPRE screen (the number of elements for making a decision what smartphone model they will purchase). The easy task was to create six elements, two for Criteria 1 and four for Criteria 2, while a difficult task was instructed to consist of 12 elements: four for Criteria 1 and eight for Criteria 2. Seventeen of 33 participants conducted the easy task.

#### Time Pressure

Time constraints for performing the decision-making task through Web-HIPRE were assigned to 18 of 37 participants, and the remaining 19 subjects were not provided with this notification. The 18 participants were informed that the reward scheme differs, depending on the time spent in completing the task. The alert was given to them that they would be paid 5,000 WON (about US $5) for completing the decision model within 1 min, 4,000 WON for 2 min after that, and 3,000 WON for longer than 2 min. However, during the postexperiment debriefing session, they were told that the notice of differential payment was a deception necessary for the experiments, and they all would receive the 5,000 WON. Those who participated in the time-constraint experiment were instructed to implement the hard task option.

### Measures

#### Visual Attention

Eye movements were detected and recorded with the Tobii X2-60 eye-tracking system, which has a 60-Hz sampling rate (SD, approximately 1 Hz: Tobii Technology, 2014). In this experiment, the visual attention patterns were presented on a screen (CRT 25 inches, 1,920 × 1,080 resolution) connected to a desktop computer that is compatible with the Tobii Studio software (v. 3.3). The accuracy and special resolution of the instrument are specified as 0.40° and 0.340°, and the total system’s latency to access data for the eye positions is less than 32 ms. The Tobii X2-60 system permits both dark and bright pupil tracking as underpinning automatic optimization. In this study, when participants performed tasks in the Web-HYPRE DSS, visual attention was measured in three AOIs using an eye-tracking tool. TFD, FC, and VC were employed as the metrics of visual attention. TFD represents the total fixed time within a specific AOI, whereas FC corresponds to the number of times that eyes are fixed on a specific AOI. VC is measured by the number of times the participant’s gaze enters the AOI (Ares et al., 2013; Pfeiffer et al., 2020). The Web-HYPRE DSS screen was divided into three AOIs: reference, task activity (from now on referred to as activity), and alternatives. The eye-tracking measures in the AOIs were not normalized for the values in any specific area. Activity is an area where participants are supposed to create elements of Criteria 1 based on their needs and come up with two elements per element of Criteria 1 in Criteria 2, for the sake of deriving the most desirable of the three alternatives. Each element of Criteria 1 must be linked to two elements in Criteria 2. The connection between elements in Criteria 2 and the alternatives is determined considering the relationship between the elements and three smartphones in the alternative area. The reference area (displayed on the screen as a reference base) is designed to be randomly located on the right or left side. Participants were instructed to refer to the hints given in the reference base (three for the easy task and six for the hard task) or rely on their ideas without consultation to the hints. Visual attention in the reference area was defined as an exploitative activity in our research model, drawing on the exploration–exploitation theory. Exploration was the value obtained by subtracting the visual attention value from the reference area from the corresponding value in the activity area since exploratory work is involved in original ideas, not an approach to improving existing knowledge or information. Participants were allowed to generate elements of the activity area (AOI-activity) in Korean or English. The Tobii program was launched with the Web-HIPRE screen open. Before starting the eye-tracking experiment, the participants’ eyes were calibrated to help fixate the three AOIs in the Web-HIPRE display. They were asked to observe the display areas by moving their pupils while keeping their body steady posture following the red dot that moves during calibration. The recording of the eye tracking continued until the decision model was completed.

#### Exploitative and Exploratory Activity

We employed the visual attention tools measured by the eye tracker and conducted a *t* test to verify that there were significant mean differences between each pair (e.g., easy vs. hard and with time constraints vs. without the option). The level of exploitative activity was defined by the values of the visual attention indices for the AOI-reference that the participants could use as a hint to perform the task; the level of exploratory activity was calculated by subtracting the visual attention value for the AOI-reference nodes from that for the AOI-activity; thus, exploration represented the extent to which subjects solved the task without help from the hint provided. Exploration was graded according to the number of criteria that participants created based on their original ideas without using the references presented (March, 1991; Laureiro-Martínez et al., 2015). On the other hand, exploitation was obtained from the visual attention values for the reference site, as they signified the extent to which the participants used general ideas, made efficient selections from the information provided, and tried to optimize their performance of a particular task (Laureiro-Martínez et al., 2015).

#### Consumer Decision-Making Creativity

In our experimental model, the CDMC criterion was based on the method that Hsiao et al. (2017) used to evaluate creativity. The detailed evaluation criteria for each activity are as follows:

Novelty: Ability to derive decision-making processes and results from his/her own ideas that are not presented by the reference nodes.
Rationality: The ability to make logical links in the decision-making process: It is evaluated by judging whether the links between elements of Criteria 1 and 2 and alternatives logically match.
Usefulness: The degree to which the practical use of the smartphone is considered. It is measured considering whether the contents given in the Criteria 1 and 2 are suitable for the overall evaluation items of the actual smartphone users.

Using the consensus assessment technique (Amabile, 1982; Althuizen and Reichel, 2016), two creativity assessment experts were invited to evaluate the outcomes of the participants’ works on Web-HIPRE. Each of the two raters assessed the three areas of creativity (e.g., novelty, rationality, and usefulness), without prior knowledge of each participant’s major, gender, and age. The raters used the five-point scale, which was anchored at 1 = “not at all” and 5 = “very agree” (Rosa et al., 2014). The total score of CDMC evaluation is in the range of 3–13 points. The interclass correlation coefficient (ICC) (Shrout and Fleiss, 1979; Baer and Oldham, 2006) of the total score showing measures of agreement among the multiple raters was 0.877 (*F* = 8.131, *p* = 0.000). CDMC was computed by averaging the scores of both raters for the three evaluation criteria. The ICCs for novelty, rationality, and usefulness were calculated as 0.775 (*F* = 4.446, *p* = 0.000), 0.782 (*F* = 4.582, *p* = 0.000), and 0.836 (*F* = 6.083, *p* = 0.000), respectively. Therefore, it is judged that there is a satisfactory agreement between the two experts.

## Results

**FIGURE 4:**
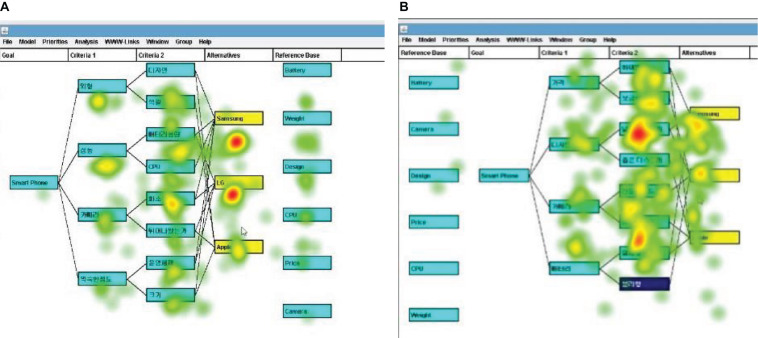
Heat maps of Web-HIPRE game activities for the hard task. **(A)** Heat map when locating the AOI-Reference on the right. **(B)** Heat map when locating the AOI-Reference on the left.

We conducted a multiple regression analysis with the predictors of task difficulty, hint location, and time constraint on exploitative (ET) and exploratory (ER) visual attentions employing three metrics such as TFD, FC, and VC. The regression models of the ET gauged by the three visual attention metrics were adopted with a relevant fitness: adjusted *R*^2^ = 0.182, *F* = 6.122, *p* < 0.001, for TFD; adjusted *R*^2^ = 0.326, *F* = 12.145, *p* < 0.001, for FC; adjusted *R*^2^ = 0.237, *F* = 8.139, *p* < 0.001, for VC (Table 2). On the other hand, for ER, only the FC regression model showed significance: adjusted *R*^2^ = 0.008, *F* = 1.196, *p* > 0.05 for TFD; adjusted *R*^2^ = 0.107, *F* = 3.761, *p* < 0.05, for FC; adjusted *R*^2^ = 0.048, *F* = 2.170, *p* > 0.05, for VC (Table 3). Task difficulty predictor showed a positive effect on ET in terms of TFD, FC, and VC visual attention metrics. That is, it suggests that if the task is difficult, people refer to and improve existing information or knowledge (exploitative activity) (TFD: *β* = 0.364, *p* = 0.002; FC: *β* = 0.415, *p* = 0.000; and VC: *β* = 0.379, *p* = 0.001) (Table 2). However, the predictive effect of task difficulty on ER activity was not verified (Table 3). Therefore, H1 was partially supported, which demonstrated that the participants engaged more ET activity when the task was harder, but the task difficulty had no effect on the ER behavior. We also hypothesized that ER activity gauged by visual attention metrics increases in the absence of time constraint and that ET activity increases because of time constraint manipulation (H2). The effect of the time-constraint variable was verified only for ER activity measured by FC metric, but not in the other five regressions. In the absence of time constraint, the ER activity in FC metric significantly increased (*β* = 0.354, *p* = 0.005; Table 3). Thus, H2 was partially supported. Further, we hypothesized that the visual attention to ET activity is more concentrated when the hint location is on the left rather than on the right (H3a), but the test result was calculated opposite to the assumption of H3a. Namely, when hint nodes were located on the right side, participants engaged in more exploitative activities; *β* = 0.311, *p* = 0.006 for TFD; *β* = 0.428, *p* = 0.000 for FC; and *β* = 0.329, *p* = 0.003 for VC (Table 2 and Figure 4). On the other hand, we hypothesized that the placement of hint nodes would not affect ER activity. As the location of the hint node has no statistical significance for the effect on ER activity (Table 3), H3b may be adopted.

**TABLE 2:** Effects of task difficulty, hint location, and time constraint on exploitative visual attention in terms of TFD, FC, and VC metrics.

Visual attention metric	Variable	Standardized coefficient	*P*-value	Adjusted *R*^2^	*F*
		*β*			
TFC	Task difficulty	0.364	0.002**	0.182	6.122**
	Time constraint	0.099	0.393		
	Hint location	0.311	0.006**		
FC	Task difficulty	0.415	0.000***	0.326	12.145***
	Time constraint	0.021	0.84		
	Hint location	0.428	0.000***		
VC	Task difficulty	0.379	0.001**	0.237	8.139***
	Time constraint	–0.047	0.677		
	Hint location	0.329	0.003**		

*N* = 70. **p < 0.01, ***p < 0.001. Significance levels are two-tailed. Note. If the hint location variable’s *β* is computed to positive, it means that the right position has a positive effect on the dependent variable. Positive *β* of task difficulty means that the hard task affects the dependent variable positively. Positive *β* of the time constraint variable means that the time constraint manipulation adversely affects the dependent variable.

**TABLE 3:** Effects of task difficulty, hint location, and time constraint on exploratory visual attention in terms of TFD, FC, and VC metrics.

Visual attention metric	Variable	Standardized coefficient	*P*	Adjusted *R*^2^	*F*
		*β*			
TFC	Task difficulty	0.183	0.155	0.008	1.196
	Time constraint	0.184	0.152		
	Hint location	–0.081	0.500		
FC	Task difficulty	0.203	0.098	0.107	3.761*
	Time constraint	0.354	0.005**		
	Hint location	–0.164	0.155		
VC	Task difficulty	0.196	0.121	0.048	2.170
	Time constraint	0.110	0.381		
	Hint location	–0.233	0.051		

*N* = 70. *p < 0.05, **p < 0.01. Significance levels are two-tailed. Note. If the hint location variable’s *β* is computed to positive, it means that the right position has a positive effect on the dependent variable. Positive *β* of task difficulty means that the hard task affects the dependent variable positively. Positive *β* of the time constraint variable means that the time constraint manipulation adversely affects the dependent variable.

We performed multiple regression employing the CATREG (optimal scaling categorical regression) routine to test the effects of exploitative and exploratory visual activities on CDMC as measured by eye-tracking tool and experts’ assessments, which depicted the results in Table 4. The CATREG routine was developed by the Data Theory Scaling System Group (DTSS) at Leiden University and is a descriptive, non-linear multivariate procedure that is available in SPSS (van der Kooij et al., 2006; Kooij, 2007; Ruiz et al., 2011). CATREG performs an “optimal scaling” regression analysis that allows simultaneous scaling of nominal and continuous variables and optimization of model fitness with various scaling levels. An optimal model fit was achieved by designating all of the independent variables as “numeric” and the dependent variable (CDMC) as “ordinal” (adjusted *R*^2^ = 0.244, *F* = 2.933, *p* < 0.05). The dependent variable was discretized to six groups employing the normal distribution option. In terms of the FC metric, exploratory activity influenced CDMC positively (*β* = 0.917, *p* = 0.002). Based on the test result shown in Table 4, H4 was partially supported.

**TABLE 4:** Effects of exploitative and exploratory activities on CDMC.

ET and ER activities	Variables (visual attention metric)	Standardized coefficient	*P*	Adjusted *R*^2^	*F*
		*β*			
ET	TFD	–0.178	0.940	0.627	3.158*
	FC	–0.761	0.356		
	VC	1.055	0.178		
ER	TFD	–0.700	0.286		
	FC	1.162	0.002**		
	VC	–0.658	0.140		

*N* = 37. *p < 0.05. **p < 0.01. Note 1. ET and ER stand for explorative and exploratory activities, respectively. Note 2. The variables are based on visual attention metrics per minute. Note 3. This regression model was analyzed with a group for the time-constraint experiment.

## General Discussion

### Key Findings and Implications of the Study

Our experiments provided the following key findings by using the eye-tracking device to measure visual attention with Web-HIPRE. Our findings contribute to the academic discipline by presenting new perspectives of visual attention, exploitation–exploration activities, and CDMC in a Web-HIPRE game environment. In particular, research on creativity in consumers’ decision-making is scarce, although its importance is not easily ignored.

First, from the experimental results of consumers’ decision-making employing the Web-HIPRE game, task difficulty affected participants’ visual attention to the exploitative activity. In contrast, time constraints had an adverse effect on exploratory activity. (1) The more difficult the task, the more likely participants were to fixate on the AOI-reference more frequently and for a longer period and (2) compared with the time constraint option manipulated by incentive, the participants’ visual attention to exploratory activity was greater in the absence of time constraints. However, the time constraint factor did not influence the exploitative activity. The results are consistent with an antecedent study by Lee and Meyer-Doyle (2017) that individuals’ exploration activities get weakened when incentivizing for their performances; namely, the exploratory activity is negatively affected by the time constraint option where the reward amount increases as the task execution time lessens.

By comparison with people who undertake easy tasks (involving less complex decisions), those with difficult tasks (more complex decisions) grapple with the problems and endeavor to come up with a creative idea that leads ultimately to a better outcome. Drawing on the study of Bang and Wojdynski (2016), people have a propensity to attend more and longer when they engage in tasks with high cognitive demand, while they attend relatively less to a simple task that requires little cognitive effort. At the individual level, exploitation is defined as behavior that is related to the selection and improvement of existing ideas designed to optimize the performance of a current task. In contrast, exploration involves departing from current capabilities and general ideas to perform new experiments and derive new ideas and outcomes (March, 1991; Laureiro-Martínez et al., 2015). Drawing on the results of the experiment that employed a Web-HIPRE game to assess creative decision-making in a mobile phone purchase, the participants tended to actively use the hints presented on the screen when engaged in a more complex problem. As the number of attributes required for decision-making increases, the decision-making process becomes more difficult. Based on our analysis of the experimental results, it is inferred that people tend to seek information by searching the surroundings more extensively in these situations.

Another finding was that exploitation was involved more when the hint nodes were placed on the right rather than on the left side of the screen, such that, when locating the reference (hint nodes) on the right side, they directed greater visual attention to the area of reference, opposite to our expectation that the left side would be more familiar to the subjects and more involved in exploitation because many websites have filters and menus on the left side. In alignment with Norman’s perceived affordance theory (Norman, 1999), influenced by Gibson’s affordance effect, which signifies that familiar information and circumstances affect human behavior (Gibson, 1979; Michaels, 1988; Bub et al., 2018), we could interpret this result that the subjects’ habit of reading information from the left side to the right side was affected. It is speculated that visual attention was higher when placing hint nodes on the right than on the left side because it would be more familiar and convenient to look at the right side than the left side of the primary information to discover a hint during the work because of the natural flow of the language interpretation that generally starts from the left to the right since they were infants (Chokron and De Agostini, 1995; de Hevia et al., 2014; Göbel, 2015). From this point of view, depending on the target group’s characteristics (i.e., habits in languages), the designs of offline advertisements or web pages can be considered by applying the affordance theory.

Table 4 depicts that CDMC is not expected to increase even if the exploitation activity enhances, whereas the visual exploratory activity positively affects CDMC. The increase in exploratory activity is found in those who have driven creative decision-making, aligning with previous research claims that individuals with the exploratory propensity produce better decision-making outcomes (Laureiro-Martínez et al., 2015; Lee and Meyer-Doyle, 2017). This result also suggests that the external information does not have a decisive influence on the creative purchasing decision (manufacturer’s explanation or advertisement), given the exploitative activity was not determined as a significant predictor.

### Implications for Marketing Practice

Our results have some interesting implications for both practice and management. First, recent trends in online shopping homepages show that sufficient information is displayed on the screen to attract consumers’ attention. This issue often confuses and imposes a cognitive overload because consumers are required to make complex decisions. In this respect, our results revealed that relevant phrases and information need to be arranged on the right side of the online shopping screen to help consumers make purchase decisions in cases in which target products require complex decision-making.

Second, it would be quite useful for companies to use creative consumers to find the existence of unsatisfactory needs remaining in the target market when preparing new products. Because creative consumers were unexpectedly able to disclose the existence of unmet needs in the target market, this insight could be useful to acquire a competitive advantage for firms in the fierce competition among companies. Therefore, companies must be prepared well to identify creative consumers.

### Limitations and Future Research Suggestions

Despite our findings, there still are limitations to this research. First, this study only employed monocultural data samples acquired from Korean student. As the application of country classification is essential for global firms such as smartphone manufacturers to set up an international marketing strategy, as Helsen et al. (1993) and Lysonski et al. (1996) proposed, a further utilization with several data samples gathered from consumers in different countries would be much helpful for field practitioners to discover hidden ethnic heterogeneity. Also, because of the single type of participant for the experiments (Korean and student), this study’s results could be limited. The results might differ if considering subjects with different age groups and other life habits in terms of work, family, language, or culture. Hence, a future study can examine cultural differences, including consumers from several backgrounds (e.g., Korean vs. US consumers and students vs. permanent employees) on similar research topics.

Next, this study focused only on the creativity of consumers. However, there are still evocatively relevant links between consumers’ creativity, innovativeness, and novelty-seeking (Hirschman, 1980), and innovative consumers tend to acquire a significantly provocative value (Kim et al., 2020a). In particular, if we consider smartphones are a collection of innovations, a future study would be necessary to take other innovativeness and novelty traits of consumers with creativity into account in order to derive more fruitful outcomes.

Also, besides various advantages of the eye-tracking experiment, there are still a few disadvantages: approximately 10% to 20% of participants cannot be examined because of contact lenses, glasses, and pupil colors (Jacob and Karn, 2003); outcomes from eye-tracking experiments could not be the only impactful measurement to discover the intention of consumers and thus might need additional evaluations (Orquin and Loose, 2013). Therefore, we suggest researchers in the field use integrated approaches such as eye tracking with functional near-infrared spectroscopy (fNIRS) or eye tracking with electroencephalography (EEG), and then it would be possible to draw many beneficial findings without facing bias from self-report surveys.

Lastly, this research used the conceptual e-commerce environment using a Web-HIPRE game to evaluate consumers’ creative decision-making in a mobile phone purchase. Hence, we suggest that this study’s results also need to be confirmed in a standard e-commerce environment in future research.

## Data Availability Statement

The datasets presented in this article are not readily available because datasets used for this study require participants’ consent before being shared with others. Requests to access the datasets should be directed to KL, kunchanglee@gmail.com.

## Ethics Statement

The studies involving human participants were reviewed and approved by the IRB of Sungkyunkwan University. The patients/participants provided their written informed consent to participate in this study.

## Author Contributions

EC designed the experiment, collected and analyzed the data, drafted and revised the manuscript. CK assisted with the experiment, analyzed the data, and drafted and revised the manuscript. KL supervised the experimental design and the data collection and revised the manuscript. All authors contributed to the article and approved the submitted version.

## Conflict of Interest

The authors declare that the research was conducted in the absence of any commercial or financial relationships that could be construed as a potential conflict of interest.

## Footnotes

1 http://hipre.aalto.fi/
